# Predictive Significance of Serum Level of Vascular Endothelial Growth Factor in Gastric Cancer Patients

**DOI:** 10.1155/2016/8103019

**Published:** 2016-08-11

**Authors:** Lu Wang, Yanli Chang, Jianjun Xu, Qingyun Zhang

**Affiliations:** Department of Clinical Laboratory, Key Laboratory of Carcinogenesis and Translational Research, Ministry of Education, Peking University Cancer Hospital & Institute, Beijing 100142, China

## Abstract

The study aims to evaluate serum VEGF expression in gastric cancer patients and investigate its relationship with clinicopathological parameters. We also examined the serum VEGF levels in GC patients having received surgery or chemotherapy treatment to assess its predictive and prognostic value as a biomarker. We enrolled 154 GC patients having not received neoadjuvant treatment and 100 healthy controls. In the treatment groups, 13 surgery patients and 15 chemotherapy patients were investigated. 42 chemotherapy patients with different chemotherapy efficacy were recruited as well. The serum VEGF was examined by ELISA. Serum VEGF level was remarkably upregulated in GC group compared with healthy group (*p* < 0.001). The serum VEGF level of GC group was significantly correlated with tumor cells differentiation degree, clinical stages, tumor infiltration depth, lymph node metastasis, and tumor size. The serum VEGF level of the 1 to 3 days after operation group was much lower than that of the preoperative group (*p* < 0.001) and the 7 days after operation group (*p* < 0.001). By contrast, serum VEGF level was decreased significantly after chemotherapy (*p* = 0.001). Importantly, serum VEGF level in PD+SD group was significantly higher compared to the PR+CR group (*p* = 0.011). Therefore, serum VEGF was a valuable biomarker in clinically monitoring the condition of GC patients.

## 1. Introduction

Gastric cancer is one of the most common cancers and is the second cause of cancer-related death worldwide [1, 2]. Gastric adenocarcinoma accounts for about 95% of gastric cancer cases, and the highest incidence countries are in Eastern Asia (e.g., Korea, Japan, and China) [3, 4]. Moreover, males are affected twice as frequently as females, and the average age of onset is between 60 and 70 years [5]. Conventional therapy options for gastric cancer include surgery, chemotherapy, radiation therapy, and combination treatments. In the early stages, the disease can often be cured by complete surgical removal of the tumors. However, patients with advanced gastric cancer indicated a poor prognosis [6]. Thus, there is the need for a better marker to identify the condition and prognosis of patients.

Angiogenesis is a process of new blood vessels formation that plays a critical role in the growth and metastasis of many solid tumors including gastric adenocarcinoma. Among multiple proangiogenic factors that participate in physiological and pathological angiogenesis, VEGF is the most important [7, 8]. VEGF can bind to VEGF receptor (VEGFR) to trigger multiple cellular signal pathways which inhibit apoptosis and stimulate survival of endothelial cells and recruit of endothelial precursor cells from the bone marrow to the sites of angiogenesis. It also plays an important role in increasing vascular permeability and inhibiting differentiation of dendritic cells [9]. Notably, serum VEGF level is higher than that of plasma. VEGF is mostly produced by tumor cells and transported by platelets. It is released into serum after platelets degranulation during blood clotting. Therefore, serum VEGF levels can better reveal the amount of VEGF produced by tumor cells and tumor burden [10].

The current study aimed to identify serum VEGF as a biomarker that may be used to monitor both surgery and chemotherapy patients during the course of the disease and investigate the clinical significance of serum VEGF level to predict chemotherapy outcomes.

## 2. Materials and Methods

### 2.1. Study Cohort

Between September 2015 and January 2016, 154 patients with histologically confirmed gastric adenocarcinoma in Peking University Cancer Hospital were enrolled as the GC group. The GC patients included 112 males and 42 females, with a mean age of 60 (range 27 to 82 years). None of the patients in this group have received neoadjuvant treatment before. Tumor staging was based on clinical information, radiologic reports, operative findings, and pathology reports. The staging was made in accordance with the TNM staging system for gastric cancer and TNM staging was done according to the American Joint Committee on Cancer (AJCC). In addition, 100 healthy controls including 70 males and 30 females, with a mean age of 40 years (range 22 to 61 years), were chosen at the Medical Examination Center of Peking University Cancer Hospital.

In order to evaluate the effects of treatment on the serum VEGF levels in gastric cancer patients, we selected 13 gastric cancer patients in the operation group and 15 patients in the chemotherapy group. Their blood samples were collected before and after treatment, respectively. No one in the operation group received chemotherapy or radiotherapy before or after surgery. Moreover, 42 gastric patients who were undergoing their chemotherapy cycles in Peking University Cancer Hospital were selected.

### 2.2. Detection of Serum VEGF

A total of 5 mL peripheral venous blood was obtained and then centrifuged to collect serum. Serum samples were stored at −80°C for further use. The serum VEGF was detected using quantitative human VEGF sandwich enzyme immunoassay kits (Jian Ping Jin Xing Biological Technology Co., Ltd., Beijing, China) following manufacturer's instructions, under standard conditions. The ELISA readings were measured at 450 nm in a microplate reader.

### 2.3. Evaluation of Chemotherapy Effect

All chemotherapy patients were assessed with CT scan after 2 chemotherapy cycles. The treatment effect was assessed based on response evaluation criteria in solid tumors (RECIST) guidelines [11]: complete response (CR): all known disease disappearing; partial response (PR): ≥30% reduction in the sum of linear tumor measurements; progressive disease (PD): at least 20% increase in the sum of target lesions and new lesions appearing; and stable disease (SD): neither enough shrinkage for PR nor enough increase for PD. CR and PR were defined as effective response while SD and PD were labelled as invalid response.

### 2.4. Statistical Analysis

Statistical analysis was performed using SPSS 19.0 for Windows. Statistical significance was measured by *p* value < 0.05. Measurement data with normal distribution was expressed as mean ± standard deviation, while median (interquartile range, IQR) was used when the data did not meet the normal distribution. Comparison between the GC group and control group and parameters that contained 2 varieties were evaluated with a Mann-Whitney *U* test. Repeated measures ANOVA was applied in continuous monitoring operation patients group while paired-samples *t*-test was used in chemotherapy patients group. Two-independent-sample *t*-test was used to evaluate the relationship between clinical progression and VEGF levels.

## 3. Results

### 3.1. Characteristics of the Gastric Cancer Patients

There were 112 (72.7%) males and 42 (27.3%) females in the GC group and the mean age of GC group was 60 years (range 27 to 82 years). The baseline parameters of gastric cancer patients were shown in Table 1. The serum VEGF levels of gastric cancer patients showed no correlations with gender, age, distant metastasis, Lauren classification, Her2, and EGFR expressions (*p* > 0.05, Table 1). The serum VEGF level of patients with poorly differentiated carcinoma was significantly higher than that of moderately differentiated and well-differentiated carcinoma (*p* = 0.012). Serum level of patients with early clinical stages (I+II) was significantly lower than that of advanced clinical stages (III+IV) (*p* = 0.004). In addition, serum VEGF level was markedly higher in patients with infiltration depth of T3 and T4 compared with that of the T1 and T2 group (*p* = 0.004). Serum VEGF level of patients who had lymph node metastasis was higher than that of patients who had no lymph metastasis (*p* = 0.036). Meanwhile, compared to the group with tumor diameter within 4 cm, the serum VEGF level of the group with tumor diameter greater than or equal to 4 cm was significantly higher (*p* = 0.028).

### 3.2. VEGF Expression in the Sera of Gastric Cancer Patients and Controls

In order to test the serum VEGF levels as the potential biomarker for early prediction of gastric cancer, we selected 154 gastric cancer patients who had never received neoadjuvant treatment before and 100 healthy controls. The ELISA results showed that the serum VEGF levels were significantly different between the GC group and the control group (145.812 (143.298) pg/mL versus 54.539 (67.355) pg/mL, *p* < 0.001, Table 2).

### 3.3. Evaluation of the Effects of Treatments on the Serum VEGF Levels in Gastric Cancer Patients 

#### 3.3.1. The Dynamic Changes of Serum VEGF Levels in the Operation Group before and after Surgery

**Figure 1:**
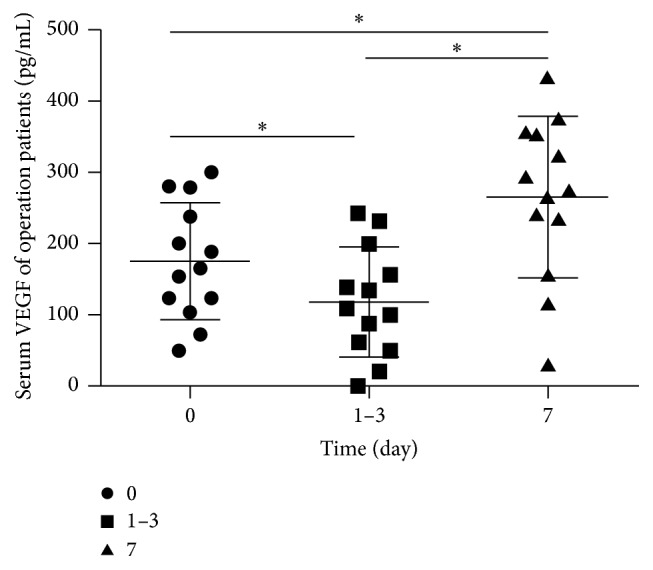
The dynamic changes of serum vascular endothelial growth factor levels in operation group with gastric cancer before and after surgery. The time of 0 days represents the time before surgery, 1–3 days represents 1 to 3 days after surgery, and 7 days represents 7 days after surgery. ^*∗*^ *p* < 0.001 by paired-samples *t*-test.

In this research, 13 gastric cancer patients were monitored to see the dynamic changes of serum VEGF levels before and after surgery within a short time. The serum VEGF levels of gastric cancer patients before surgery and 1 to 3 days and 7 days after surgery were 175.712 ± 81.329 pg/mL, 117.797 ± 76.022 pg/mL, and 266.119 ± 112.218 pg/mL, respectively. The serum VEGF levels differed significantly among the three groups (*F* = 29.002, *p* < 0.001, Table 3). The serum VEGF level of the group with 7 days after surgery was significantly higher compared to that of the preoperative group (*p* < 0.001, Figure 1) and the group with 1 to 3 days after surgery (*p* < 0.001). Moreover, the serum VEGF level of preoperative group was much higher than that of the group with 1 to 3 days after surgery (*p* < 0.001).

#### 3.3.2. The Changes of Serum VEGF in the Chemotherapy Group before and after Chemotherapy

In order to test the VEGF level as a biomarker possibility for prediction of chemotherapy efficacy, 15 patients in the chemotherapy group were chosen in order to compare the serum VEGF levels before and after chemotherapy. The serum level of VEGF was 207.740 ± 137.912 pg/mL before chemotherapy and 112.530 ± 67.124 pg/mL after chemotherapy, demonstrating a sharp decrease after chemotherapy (*t* = 4.310, *p* = 0.001, Table 4).

#### 3.3.3. Serum VEGF Level as a Prognostic Marker in Chemotherapy Patients of Gastric Cancer

Since the remarkable upregulated expression of VEGF during the development of GC, the diagnostic significance of VEGF was also necessary to explore. 42 gastric cancer patients who were undergoing their chemotherapy treatment in Peking University Cancer Hospital were enrolled in the group in order to compare serum VEGF levels of different chemotherapy efficacy. They were categorized into stable-disease- (SD-) plus-progressive-disease (PD) group and complete-response- (CR-) plus-partial-response (PR) group based on the results of CT scans obtained two cycles (one cycle contained 21 days) after chemotherapy. 15 cases were identified as effective and 27 cases as invalid. The serum levels of the PD+SD group and the PR+CR group were 156.733 ± 101.262 and 79.364 ± 66.408 pg/mL, respectively. Significant differences were observed in the VEGF levels between the PD+SD group and the PR+CR group after chemotherapy (*t* = 2.652, *p* = 0.011, Table 5).

## 4. Discussion

In 1971, Folkman and coauthors suggested that tumor growth and metastatic spread may depend on the degree of vascularization [12]. Angiogenesis, the formation of new blood vessels from existing vasculature, is an important process in many malignancies including gastric cancer [13]. Many studies have shown that VEGF played a key role in angiogenesis in gastric cancer among multiple proangiogenic factors [14–16]. The serum assay for VEGF using ELISA can be frequently and easily performed, because it is a noninvasive method compared to surgically obtained tissue materials, which might make it useful in monitoring the course of disease or response to treatment.

In the present study, serum VEGF levels were higher in GC patients than healthy controls and high serum VEGF levels were correlated with poorly differentiated tumors, advanced clinical stages, locally advanced T stages, lymph node metastasis, and larger tumor sizes. The results were consistent with the findings of previous studies [17–19]. However, Fushida et al. found serum VEGF levels were also significantly correlated with peritoneal metastasis and malignant ascites in gastric cancer [20]. In addition, Oh et al. reported a significant correlation between the serum level of VEGF and Lauren's classification [21]. So far, mostly single angiogenic factor either in blood or in tumor tissue was analyzed in limited patient numbers. This may be one of the reasons why studies revealed inconclusive results.

Previous studies showed that serum VEGF levels decreased after the completion of treatment in patients with resected tumors [19, 22]. However, dynamic changes of serum VEGF levels in operation patients were monitored in the present study. The serum VEGF levels decreased 1 to 3 days after surgical removal but significantly increased 7 days after surgery compared to preoperative levels. Radical resection possibly resulted in a sharp decline of VEGF levels within a short time. However, serum VEGF levels appeared to increase maybe due to the healing of operative incision or existence of a pathway that could produce VEGF but is not dependent on tumor tissues. The same changes of serum VEGF levels were also found in patients with non-small cell lung cancer [23].

In addition, serum VEGF levels were significantly decreased after chemotherapy. Oh et al. also reported that the median level of serum VEGF was decreased after FOLFOX chemotherapy [21]. With respect to medically treated gastric cancer patients, Kitamura et al. found a decrease in the serum VEGF levels after partial response by chemotherapy; the patients who had disease progression after chemotherapy showed an increase in VEGF levels [24]. We compared VEGF levels in a different chemotherapy efficacy group, which could predict chemotherapy response of GC cancer patients. Serum VEGF levels of chemotherapy patients with PD and SD were much higher than those with PR and CR.

A recent meta-analysis of VEGF-A expression in gastric cancer showed that VEGF-A overexpression was associated with poor overall survival (OS) (hazard ratio [HR] = 1.57; 95% confidence interval [CI], 1.30–1.84) and disease-free survival (DFS) (HR = 1.85; 95% CI, 1.39–2.32) [13]. VEGF has become a leading therapeutic target for antiangiogenic use in the treatment of cancer [9, 25]. Various kinds of antiangiogenic agents which inhibit VEGF and VEGFR have been developed and discovered gradually, including antibodies, ribozymes, and small molecule inhibitors. Ramucirumab, an antibody to the VEGF receptor 2 (VEGRF2), improved OS of GC patients compared with the best supportive care in a second-line setting [26]. A recent study found that CRMP4 expression mediated by the activation of VEGF signaling facilitated gastric tumor growth and metastasis, which may have clinical implications associated with a reduced survival rate in gastric cancer patients [27].

Interestingly, in the chemotherapy group we found that 8 patients were with peritoneal carcinomatosis and/or retroperitoneal lymph node metastasis and 7 patients with hematogenous metastasis including 4 with metastasis to the liver, 2 to the lung, and 1 to the bone. Although with a few samples studied, serum VEGF levels were still significantly different between the peritoneal carcinomatosis and the hematogenous metastasis (97.706 ± 44.225 pg/mL versus 273.921 ± 147.375 pg/mL, *p* < 0.05). The patients with hematogenous metastasis may present higher serum VEGF levels. Scartozzi et al. analyzed multiple SNPs in the VEGFs, VEGF receptors, and integrins genes in pT4a resected gastric cancer patients. They demonstrated that the AC genotype of rs699947 (VEGFA) independently correlated with hematogenous metastases, while the AA genotype of rs2269772 (ITGA) independently correlated with peritoneal-only diffusion [28]. In addition, VEGFA genotyping may help to determine clinical outcome in metastatic gastric cancer patients receiving platinum-based first-line chemotherapy. VEGF-A rs25648 genotyping may indicate patients unlikely to benefit from first-line chemotherapy and potential candidates for alternative therapy choices [29]. Thus, detection of genotyping for specific VEGFA and integrin genes may help clinicians assess the risks of metastatic process and select better therapeutic strategies for patients. In contrast, ELISA for serum VEGF level is more suitable for diagnosis and monitoring the progression of the disease. Combination of the two detection methods would allow clinicians to better assess the status of the disease and improve the treatment efficacy. We can also choose the proper methods to match the special goal in clinical practice.

However, there are some limitations in our study. Because of the small groups of patients enrolled into this study, further large collaborative studies are necessary to confirm our results. In addition, chemotherapy regimens of chemotherapy patients should be classified to better observe the response to different chemotherapy drugs. A more in-depth research may be needed to clarify the issue.

In conclusion, serum VEGF levels might be correlated with biological characteristics, such as differentiation degree of tumor cells, clinical stages, depth of tumor infiltration, lymph node metastasis, and tumor size. Moreover, in our current study, the serum VEGF levels of surgery and chemotherapy patients with gastric cancer were examined, respectively. In surgery patients, the dynamic changes of serum VEGF levels decreased first and then increased within a short time after operation. We also suggest that the serum VEGF levels may be useful to predict the response of gastric cancer patients who receive chemotherapy treatment.

## Figures and Tables

**Table 1:** Correlation of serum VEGF with clinical indicators in patients with gastric cancer (median (IQR)).

Parameters	Number	VEGF (pg/mL)	*p* value
Gender			
Male	112 (72.7%)	151.686 (143.640)	0.942
Female	42 (27.3%)	134.643 (154.517)	
Age			
≤60	70 (45.5%)	130.314 (140.753)	0.056
>60	84 (54.5%)	163.089 (163.860)	
Differentiation degree			
Low	92 (59.7%)	171.181 (158.886)	0.012
Moderate	58 (37.7%)	116.824 (104.379)	
High	4 (2.6%)	163.360 (179.338)	
Clinical stages			
I+II	84 (54.5%)	121.702 (138.479)	0.004
III+IV	70 (45.5%)	173.823 (146.996)	
Depth of infiltration			
T1+T2	59 (38.3%)	110.770 (99.111)	0.004
T3+T4	95 (61.7%)	171.692 (158.016)	
Lymph node metastasis			
No	60 (39.0%)	122.274 (112.414)	0.036
Yes	94 (61%)	171.669 (149.365)	
Distant metastasis			
No	138 (89.6%)	145.812 (148.228)	0.854
Yes	16 (10.4%)	153.702 (113.545)	
Lauren			
Intestinal	64 (41.6%)	154.309 (149.211)	0.651
Diffuse	43 (27.9%)	135.712 (145.44)	
Mixed	46 (29.9%)	155.912 (171.842)	
Unknown	1 (0.6%)	149.933	
HER2			
Negative	70 (45.5%)	149.126 (137.971)	0.397
Positive	74 (48.1%)	137.630 (155.948)	
Unknown	10 (6.4%)	151.686 (105.018)	
EGFR			
Negative	7 (4.5%)	99.352 (402.412)	0.708
Positive	127 (82.5%)	140.213 (143.821)	
Unknown	20 (13.0%)	156.699 (108.166)	
Tumor size			
≤4 cm	89 (57.8%)	129.478 (141.868)	0.028
>4 cm	49 (31.8%)	169.255 (155.814)	
Unknown	16 (10.4%)	130.409 (120.659)	

Total	154 (100%)		

**Table 2:** Comparison of serum VEGF levels between patients with gastric cancer (GC group) and healthy subjects (control group) before chemotherapy (median (IQR)).

Group	Number	VEGF (pg/mL)	*p* value
GC group	154	145.812 (143.298)	<0.001
Control group	100	54.539 (67.355)	

**Table 3:** Continuous monitoring of serum VEGF levels in patients with gastric cancer before and after operation ($\overset{-}{x}\pm s$).

Group	Number	VEGF (pg/mL)			*F*	*p* value
		0 days	After 1–3 days	After 7 days		
Operative patients	13	175.712 ± 81.329	117.797 ± 76.022	266.119 ± 112.218	29.002	<0.001

**Table 4:** The changes of serum VEGF in patients with gastric cancer before and after chemotherapy ($\overset{-}{x}\pm s$).

Group	Number	VEGF (pg/mL)		*t*	*p* value
		Before chemotherapy	After chemotherapy		
Chemotherapy patients	15	207.740 ± 137.912	112.530 ± 67.124	4.310	0.001

**Table 5:** Comparison of serum VEGF levels in patients with gastric cancer between different chemotherapy efficacy groups ($\overset{-}{x}\pm s$).

Efficacy	Number	VEGF (pg/mL)	*t*	*p* value
PD+SD	27	156.733 ± 101.262	2.652	0.011
PR+CR	15	79.364 ± 66.408		

PD: progressive disease, SD: stable disease, PR: partial response, and CR: complete response.
